# Study on adsorption and desorption of ammonia on graphene

**DOI:** 10.1186/s11671-015-1060-7

**Published:** 2015-09-16

**Authors:** Zhengwei Zhang, Xinfang Zhang, Wei Luo, Hang Yang, Yanlan He, Yixing Liu, Xueao Zhang, Gang Peng

**Affiliations:** College of Science, National University of Defense Technology, Changsha, 410073 China

**Keywords:** Graphene gas sensor, DFT, Ammonia, Adsorption and desorption

## Abstract

The gas sensor based on pristine graphene with conductance type was studied theoretically and experimentally. The time response of conductance measurements showed a quickly and largely increased conductivity when the sensor was exposed to ammonia gas produced by a bubble system of ammonia water. However, the desorption process in vacuum took more than 1 h which indicated that there was a larger number of transferred carriers and a strong adsorption force between ammonia and graphene. The desorption time could be greatly shortened down to about 2 min by adding the flow of water-vapor-enriched air at the beginning of the recovery stage which had been confirmed as a rapid and high-efficiency desorption process. Moreover, the optimum geometries, adsorption energies, and the charge transfer number of the composite systems were studied with first-principle calculations. However, the theoretical results showed that the adsorption energy between NH_3_ and graphene was too small to fit for the experimental phenomenon, and there were few charges transferred between graphene and NH_3_ molecules, which was completely different from the experiment measurement. The adsorption energy between NH_4_ and graphene increased stage by stage which showed NH_4_ was a strong donor. The calculation suggested that H_2_O molecule could help a quick desorption of NH_4_ from graphene by converting NH_4_ to NH_3_ or (NH_3_)n(H_2_O)m groups, which was consistent with the experimental results. This study demonstrates that the ammonia gas produced by a bubble system of ammonia water is mainly ammonium groups of NH_3_ and NH_4_, and the NH_4_ moleculars are ideal candidates for the molecular doping of graphene while the interaction between graphene and the NH_3_ moleculars is weak.

## Background

Ammonia detection has a great significance in many areas, such as environmental protection and industrial inspection. Furthermore, ammonia detection has good prospects in medicine diagnose. For instance, measurements of exhaled ammonia may differentiate between viral and bacterial infections in lung diseases to justify the use of antibiotics [1], and ammonia detection may be used to indirectly measure urea levels for renal disease monitoring [2]. Identifying these signaling metabolites (disease markers) and measuring them in trace concentrations is not a trivial problem, and the low concentrations of analyte molecules presents a major challenge, along with the specificity to a given analyte. Recently, low-dimensional materials used for gas detection has become a trend [3, 4], it has been reported that it is possible to use graphene as a gas sensor with high sensitivity and high accuracy for detecting ammonia groups [5, 6]. Graphene is considered to be an excellent kind of sensor material due to its following properties: (i) graphene is a single atomic layer of graphite with a larger specific area than any other materials, which maximizes the interaction between the surface dopants and the adsorbates; (ii) as a kind of special material with zero bandgap, graphene has a extremely low Johnson noise [7], for which a slight change of carrier concentration can cause a notable variation of electrical conductivity; (iii) graphene has limited crystal defects, which ensures a low level of excess noise caused by thermal switching [7]. F. Schedin et al.’s study showed that micrometer-sized sensors made from graphene were capable of detecting individual gases, and the study found out that the changes in graphene conductivity during chemical exposure were quantized, with each event signaling adsorption or desorption of a single NH_3_ molecule, and proved that NH_3_ was acting as a strong donor for graphene and the desorption was relatively difficult [5]. Hugo E Romero et al. considered that the slow NH_3_ desorption rate from supported graphene FETs was consistent with a Fickian diffusion process of molecules in the SiO_2_/graphene interface [8]. Lakshman K. Randeniya et al. has developed a new method by adding the flow of water-vapor-enriched air at the beginning of the recovery stage to realize a rapid desorption, and researchers believe that shifts in the substrate defect states of graphene caused by the water adsorption can weaken the interaction between NH_3_ and defective graphene [9]. However, O. Leenaerts et al. investigated the adsorption of NH_3_ on graphene substrate with first-principle calculations, and the results illustrated a very small adsorption energy (31 meV) and a small charge transfer (0.027 ± 0.001 electrons per molecule at pristine sites) from NH_3_ to graphene [6].

These explanations revealed the truth from some degree, but some of these explanations are still not theoretically grounded or lack experimental verifications. Therefore, it is hard to treat them as a fully complete conclusion. In this work, we studied the adsorption and desorption of ammonia groups on graphene theoretically and experimentally. Our study demonstrates that the interaction between ammonia groups NH_4_ and graphene is much stronger than that of ammonia groups NH_3_. We take the view that hydrogen atom plays an important role in the adsorption between NH_3_ and graphene, and we find that H_2_O helps NH_4_ to take off one hydrogen atom and achieves a rapid desorption from graphene sheets.

## Methods

**Fig. 1:**
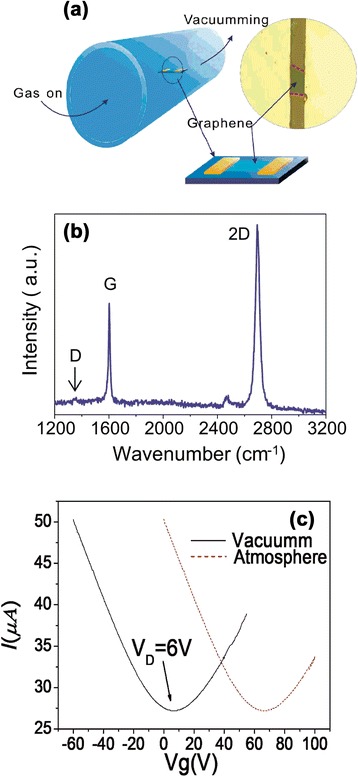
The structure of the gas sensor and a series performance tests. **a** Gas detection device and graphene-FET sensing device. A graphene sheet bridges the source and the drain electrodes, which closes the circuit. **b** Raman spectrum of the graphene transferred from highly oriented pyrolytic graphite, indicating that the transferred graphene is a single layer and the graphene layer is not damaged during the transfer (no D peak). *a.u.* arbitrary units. **c** *I*–*V*_*g*_ curve (with *V*_*ds*_ = 0.05 V) in air (*red line*) and in vacuum after 20-h vacuum treatment (*black line*)

A pristine graphene was first prepared by mechanical exfoliation (repeated peeling) of small mesas of highly oriented pyrolytic graphite, which was then transferred to highly p++ doped silicon wafer with a thermally formed SiO_2_ (300 nm) layer working as the sensors’ bottom gate through a dry transfer procedure [10, 11]. The number of layers (*N*) in a graphene film was determined with micro-Raman scattering using the shape of the 2D Raman band at ~2700 cm^−1^ and the intensity of the Raman-active G-band scattering at ~1585 cm^−1^ by a LabRAM XploRA with 532-nm excitation and low power which results was confirmed as a single-layer graphene. Then, the Cr(5 nm)/Au(100 nm) electrodes, used as the source and the drain electrodes of graphene-FET with channel width of 10 μm (Fig. 1a), were fabricated by an e-beam lithography process followed by an e-beam metal deposition process. To conveniently measure the gas-sensing properties, the electrodes of the sensors were bonded to a custom socket with gold wires. The electrical conductance, transfer characteristic, and time response of the graphene FET sensor were measured with a Keithley 2400 sourcemeter and a voltage source (±100 V). The sensing performances of as-fabricated sensing devices were characterized under practical conditions (i.e., room temperature and atmospheric pressure) against low concentration of ammonia groups produced by a bubble system of ammonia water [9]. A sensor chamber with an electrical feed through was used to house the sensing device for gas-sensing characterizations (Fig. 1a). Variations in the electrical conductance of graphene sheets were monitored by simultaneously applying a low constant DC voltage (0.05 V) and recording the corresponding change in current passing through the device when the device was exposed periodically to H_2_O or NH_3_-laden high-purity argon and vacuum environment (ultimate vacuum, ~1 Pa). A sensing test cycle typically consists of three consecutive steps that include exposures of the device to (i) vacuum environment to record a base value of the sensor conductance, (ii) target gas to register a sensing signal, and (iii) vacuum environment for the sensor recovery. All the electrical measurements were carried out at room temperature. Five different sensors for the same process were measured which showed similar behaviors except the magnitude of conductance (the difference is less than 10 %) and each measurement was repeated for at least five times.

Theoretically, the density functional theory (DFT) calculations were performed with CASTEP [12]; the generalized gradient approximation (GGA) was used to deal with the exchange and correlation term. It is well known that the local density approximation (LDA) is normally inaccurate in describing the van der Waals-like interaction, and the advantage of GGA over LDA in this work is that the GGA will not lead to a strong bonding of the molecules as LDA does [13]. A plane wave basis set with a cutoff energy of 800 eV and pseudopotentials of the Troullier–Martins type and non-spin-polarized calculations were used in this study. The total system consisted of a 3 × 3 graphene supercell (18 C atoms) with a single molecule adsorbed and a distance of 16 Å between adjacent graphene layers. The Brillouin zone integration was performed within the Monkhorst–Pack scheme using 5 × 5 × 1 k points [14]. For the calculation of the density of states (DOS), a 20 × 20 × 1 Monkhorst–Pack grid and a Gaussian smearing of 0.05 eV were used [15].

## Results and discussion

The pristine graphene was prepared by mechanical exfoliation, and the Raman measurement was applied to study the layer numbers of graphene in room temperature under the atmosphere. In Fig. 1b, no D peak (1350 cm^−1^) was observed in graphene indicating that the quality of the graphene was maintained during the transfer, and the ratio of intensity G peak and 2D peaks was about 0.5, which was a typical value of single-layer graphene. The basic electrical properties of bottom-gated bilayer graphene FETs were first studied in the atmospheric environment. We found that the Dirac peak was about 60 V (*V*_*D*_ = 60 V) (Fig. 1c red line), which illustrated that this shift was attributed to an unintentional doping of the films by absorbing water and gas molecules or other impurity substance from the supporting substrate [16, 17]. Particularly, we found that the graphene film could return to the intrinsic state roughly after 20 h of vacuum treatment (<1 Pa), and then the Dirac peak was decreased to about 6 V (*V*_*D*_ = 6 V). Prolonged vacuum treatment was capable of removing the adsorbed air molecules strongly. The total amount of effective charged impurities can be simply estimated by the amount of Dirac voltage shift, *V*_*D*_, using the following expression [16]:

1 $$ {N}_{\mathrm{imp}}={C}_{gs}\times {V}_D/e $$

where *C*_*gs*_ is the capacitance of the SiO_2_ between the graphene and the silicon substrate (the typical value is 11.5 nF/cm^2^ for 300 nm SiO_2_ in our case) and *e* is the unit charge. From Fig. 1c, we can extract the Dirac voltage shift and then calculate the corresponding amount of charged impurities by expression (1). The density of charged impurities is as high as 4.3 × 10^12^/cm^−2^ in the air, while that is about 0.43 × 10^12^/cm^−2^ in the vacuum, with the corresponding Dirac voltage point decreased from 60 to 6 V. Moreover, for each trace in Fig. 1c, we can extract the slope of linear region (*K*_*h*_ and *K*_*e*_) from which one can extract the mobilities for holes (electrons) according to [18]:

2 $$ \mu =\left(L/W\right)\kern0.75em \times \kern0.75em 1/{C}_{gs}/{V}_{SD}\kern0.5em \times \kern0.75em \frac{\partial I}{\partial {V}_g}=\frac{C\kern0.5em  \times K}{V_{SD}} $$

where *C* is a constant related to electrode geometry structure and dielectric material of the device, *K* is the slope of linear region (*K*_*h*_ for hole, *K*_*e*_ for electron), and *μ* is the mobility (*μ*_*h*_ for hole and *μ*_*e*_ for electron). From the above expression, the mobilities of the hole and electron were almost same and which value was about 2000 cm^2^(V•S)^−1^.

**Fig. 2:**
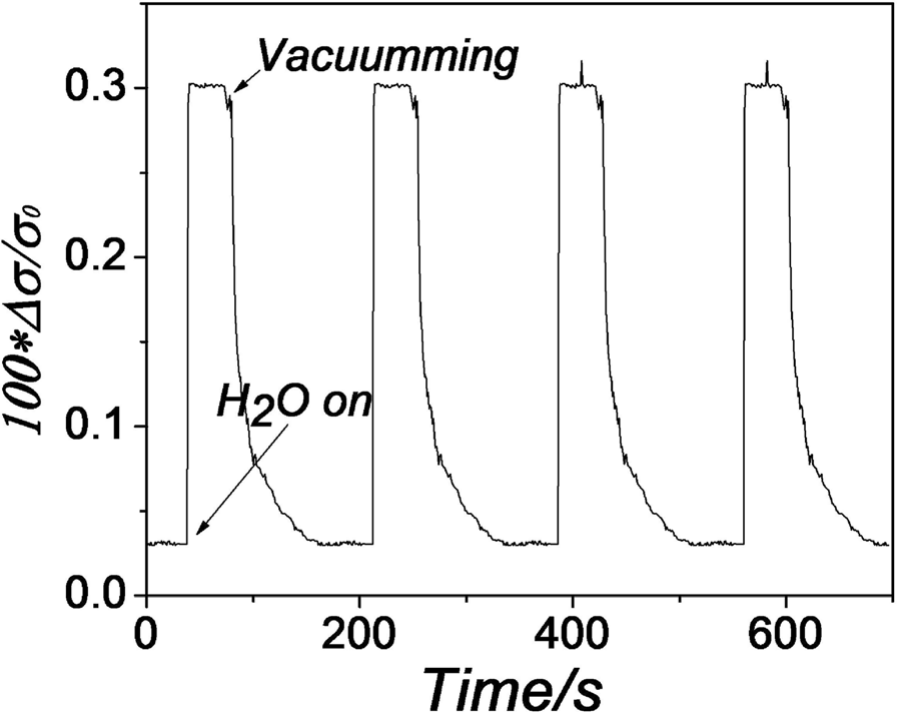
Time response of graphene conductance to water vapor. The time response of graphene gas sensor to water vapor (the relative humidity was 85 %) given in dimensionless units of 100 × ∆σ/σ_0_ where σ_0_ stands for the conductance of the graphene sensor and ∆σ is the change in conductance due to H_2_O flow

The response of graphene gas sensor to high-purity argon (higher than 99.99 %) was first measured and there was no obvious change in conductance. Then, the time response of the sensor’s conductance to a mixture gas (the relative humidity is ~85 %) by diverting dry argon air through a water bubbler bottle was measured, and the results were shown in Fig. 2. Positive values of ∆σ were consistent with the theoretical and experimental studies which suggested H_2_O is an acceptor (hole doping) molecule [6]. When the flow of H_2_O was turned off and evacuated within about 5 s, the conductance quickly decreased toward its baseline in 1 min, which illustrates that the force between graphene and water molecules is relatively weak [5].

**Fig. 3:**
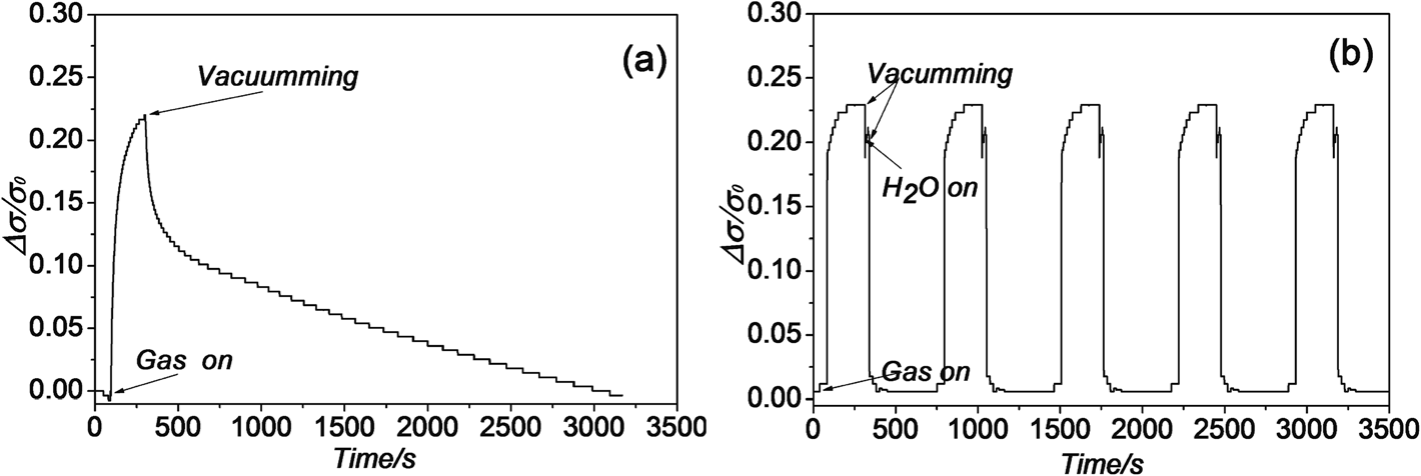
Time response of graphene conductance to 5 ppm ammonia in argon. **a** A single 2-min exposure and spontaneous recovery in vacuum treatment and **b** five consecutive 2-min exposures where water-vapor-enriched argon air was used to achieve quick desorption between exposures

The conductance of the gas sensor increased ~22.5 % when exposed to 5 ppm ammonia, shown in Fig. 3a. As is demonstrated in Fig. 3a, it took more than 1 h to reach the baseline value for the recovery stage. Fig. 3b shows a series of consecutive cycles where the recovery times were reduced to less than 2 min by adding the flow of water-vapor-enriched air at the beginning recovery stage as follows. When the ammonia gas was turned off and pumped out of chamber, water-vapor-enriched air was sucked into the chamber immediately and kept in the chamber for 1 min, then a vacuum was used to desorb all the gas. The conductance changes and the response shape profiles were extremely reproducible. These results confirmed the complete and rapid removal of ammonia from graphene sheets by water vapor [9].

**Fig. 4:**
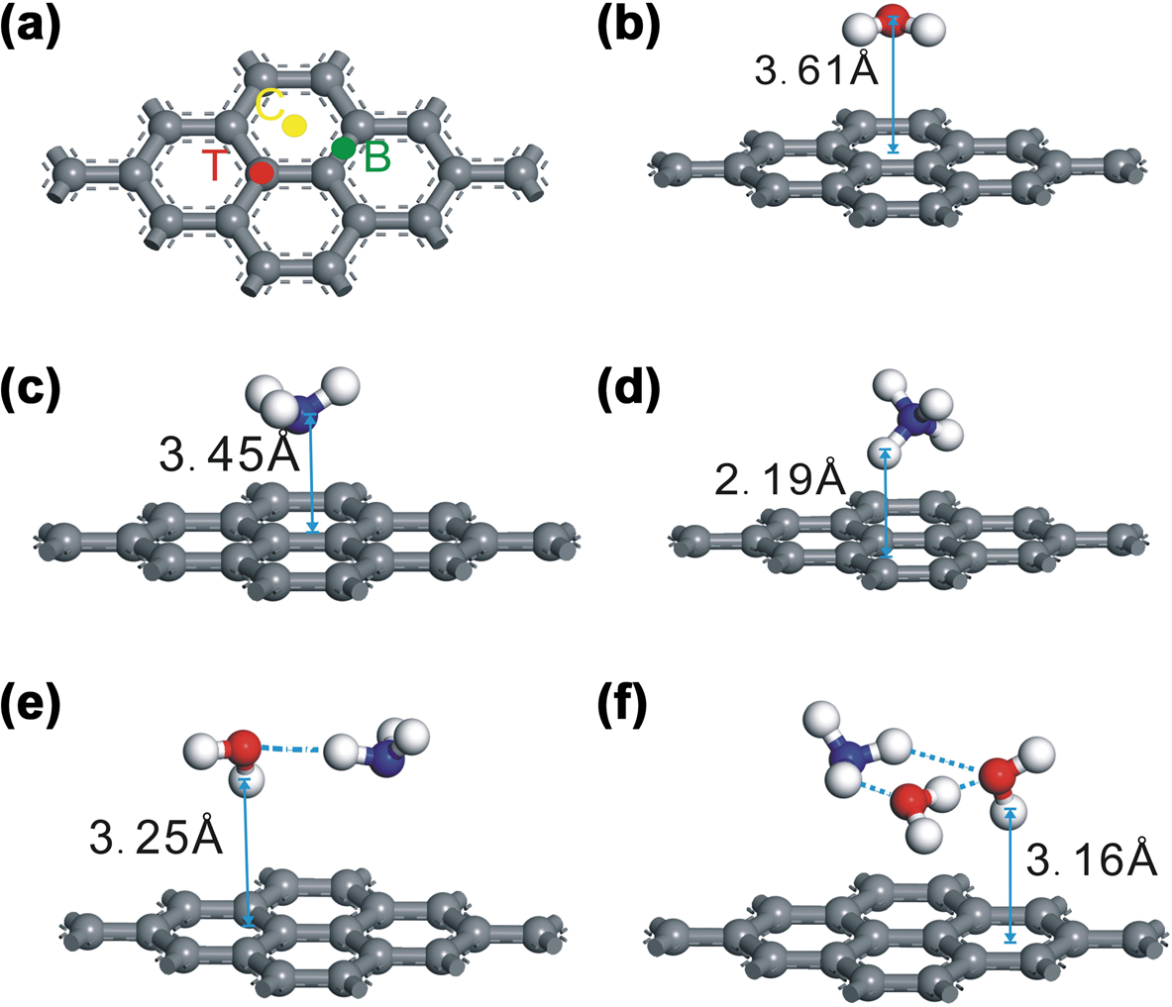
Gas on graphene. Supercell (3 × 3) of graphene adsorbed all kinds of molecules and three adsorption sites are considered (**a**). Configuration of the H_2_O (**b**), NH_3_ (**c**), NH4 (**d**), NH_3_•H_2_O (**e**), and NH3•2H_2_O (**f**) molecular on graphene sheets, all of the structures after optimization

Theoretical calculations on isolated defect-free graphene predicted that NH_3_ molecules should physisorb to the film without significant change of the band structure, and only a slight charge transfer of 0.03 − 0.04 e per adsorbed NH_3_ molecule was predicted to occur [6]. It seems that theoretical calculations are inconsistent with our experiment results. To fully clarify the mechanism of the adsorption and desorption between ammonia and graphene sheets, it is important to understand the interactions between the graphene surface and the adsorbate molecules. The first-principle calculations were performed based on DFT which has been successfully applied to study the molecular adsorbates on single-walled (carbon) nanotubes (SWNT) and graphene sheets [6, 19]. In our calculation, the molecules were first placed in high symmetry positions (C, B, and T sites) and then relaxed [6]. The configurations of the molecule-graphene system are shown in Fig. 4. Generally, larger absolute value of the adsorption energy (*E*_*a*_) means stronger binding between molecule and graphene. The *E*_*a*_ between the gas molecule and graphene is defined as

$$ {E}_a={E}_{\mathrm{system}}-{E}_{\mathrm{graphene}}-n{E}_{\mathrm{molecule}} $$

where *E*_system_ is the total energy of the optimized equilibrium configuration of the graphene and the adatom; *E*_graphene_ is the total energy of the isolated graphene; *E*_molecule_ is the total energy of the corresponding adsorption molecules in its ground state; *n* is the number of gas molecules in the system.

The following orientations of the molecule with respect to the graphene surface was studied in our calculations, for H_2_O molecule starting from the O atom, the H-O bonds pointing parallel to the graphene surface (P) [5], and two orientations of the ammonia molecule were investigated, one with the H atoms pointing away from the surface (U) and the other with the H atoms pointing to the surface (D). The calculation results for the optimized structures were summarized in Tables 1 and 2. From Table 1, the adsorption energy was about 26–29 meV per H_2_O-graphene group for different H_2_O positions with sheet-adsorbate distances of 3.6 Å (Fig. 4b), and only 0.01–0.02 e charges were transferred from graphene to the H_2_O molecule. The results suggest that H_2_O acts as an acceptor on graphene which is in accordance with our experiment. From Table 2, the adsorption energy in NH_3_-graphene group was small (31–45 meV) as well as that in H_2_O-graphene group, and the charge transfer, however, was solely determined by the orientation of the NH_3_ molecule. There was a slight charge transfer of 0.02 e from the molecule to the graphene surface in the U orientation and a extremely slight charge transfer (0.01 e) from graphene to NH_3_ molecule in the D orientation, and the bottom atom of the physisorbed molecule was located 3.4–3.6 Å (Fig. 4c) above the graphene layer. The calculation results indicated weak adsorption ability between NH_3_ molecule and graphene in both orientations, which is difficult to explain the strong adsorption and the donor character as observed experimentally.

**Table 1:** The adsorption energy *E*_*a*_ and the charge transfer *∆Q* from H_2_O to graphene with three different geometries

Position	Orientation	Distance/Å	*E*_*a*_ (H_2_O)/meV	*∆Q*
B	P	3.62	26.49	−0.01 e
C	P	3.61	29.32	−0.02 e
T	P	3.61	26.54	−0.01 e

**Table 2:** The adsorption energy *E*_*a*_ and the charge transfer *∆Q* from NH_3_ to graphene with six different geometries

Position	Orientation	Distance/Å	*E*_*a*_ (NH_3_)/meV	*∆Q*
B	U	3.40	31.44	0.02 e
C	U	3.49	45.53	0.02 e
T	U	3.66	38.04	0.02 e
B	D	3.46	39.45	−0.01 e
C	D	3.45	48.87	−0.01 e
T	D	3.45	39.42	−0.01 e

These results were in agreement with those already reported in previous studies on the binding energies of physisorbed NH_3_ molecules on graphene [5, 6]. All of these results showed that ammonia molecules NH_3_ are not good candidates for effective molecular doping of graphene. Obviously, there are other chemical groups that physisorb more strongly on graphene sheets and shift the Fermi energy inside the valence or conduction bands. Bradley et al. found that vacuum-degassed SWNT-FETs were insensitive to NH_3_; they suggested that to detect NH_3_ gas in the FET response, one should dissolve the NH_3_ in a H_2_O monolayer which forms on the nanotube FETs under ambient lab conditions. They proposed that the NH_3_ molecule in this environment could become a cation with the charge compensated from the SWNTs, i.e., acting as an *n*-dopant [20]. In fact, hydrogen is an ordinary impurity in electronic devices [21, 22], playing an important role in many reactions between physisorbed NH_3_ molecules and hydrogen adatoms to generate chemical species that have stronger interactions with the graphene layer. Specifically, an isolated H impurity on graphene can be captured by an NH_3_ molecule in an exothermic reaction that releases 1.00 eV of energy [15].

Furthermore, results for physisorbed NH_4_ species on a defect-free graphene layer was calculated in different graphene positions and a special orientation [15] (two of the NH_4_ hydrogen atoms pointing toward the graphene sheets, while the other two hydrogen species are on the other side of the ammonium group, see Fig. 4d). From Table 3, we noticed that the NH_4_ molecule were adsorbed on graphene in a stronger manner, and the interaction between dangling bonds of graphene and NH_4_ was very strong and partial electron transfer occurs from graphene. The adsorption energy was 528–644 meV which was much larger than that of NH_3_ and H_2_O adsorbed in graphene sheets, and a huge charge transfer (0.19–0.54 e) from NH_4_ to graphene proved that physisorbed NH_4_ acted as a stronger donor for the basal plane of graphene, and the bottom atom of the NH_4_ was located 2.19 Å (Fig. 4d) above the graphene layer. There was a tight connection between them, and the results were in accordance with our experiment results.

**Table 3:** The adsorption energy *E*_*a*_ and the charge transfer *∆Q* from NH_4_ to graphene with three different geometries

Position	Distance/Å	*E*_*a*_ (NH_4_)/meV	*∆Q*
B	2.86	528.06	0.19 e
C	2.19	644.29	0.54 e
T	2.21	628.94	0.34 e

It is well known that there exists complicated chemical equilibrium in ammonia-water mixtures, and the presence of NH_4_^+^ and OH^−^ ions in water solutions is a well-established fact that meets the following reaction:

$$ {\mathrm{NH}}_3 + {\mathrm{H}}_2\mathrm{O}\iff \mathrm{N}\mathrm{H}{4}^{+} + {\mathrm{OH}}^{-}\;{\mathrm{K}}_{\mathrm{b}}=\frac{\left[{\mathrm{NH}}_4^{+}\right]\left[{\mathrm{OH}}^{-}\right]}{\left[{\mathrm{NH}}_3\right]}, $$

and the experimental equilibrium constant *K*_*b*_ for this reaction at room temperature is 1.77 × 10^−5^; therefore, the concentration of NH_4_^+^ and OH^−^ in water solutions, although extremely small, is about 4.21 × 10^−3^ mol/dm^3^.

In fact, ammonia water exists in the form of clusters by hydrogen bonding [23]. Up to now, among all the possibilities, the only two which are experimentally supported are NH_3_•H_2_O and NH_3_•2H_2_O [24]. We chose these two typical groups for the calculation. Table 4 shows the construction of the clusters adsorption on the graphene sheets, and the orientations of the groups are parallel to the graphene surface (shown in Fig. 4e, f). Ignoring the adsorption site, we can find the adsorption energy is 43–73 meV, which is lower than that of NH_4_ adsorbed in graphene with a reduction of one order of magnitude, and the charge transfer from clusters to graphene sheets is close to zero. The distance between the cluster and the graphene layer is about 3.16–3.25 Å (Fig. 4d), which illustrates that the ammonia-water groups have weak adsorption with graphene sheet, and that they can be desorbed from graphene easily.

**Table 4:** The adsorption energy *E*_*a*_ and the charge transfer *∆Q* from NH_3_•H_2_O and NH_3_•2H_2_O to graphene

Distance/Å	*E*_*a*_ (NH_3_•H_2_O)/meV	*∆Q*
3.25	43.70	0.01 e
Distance/Å	*E*_*a*_ (NH_3_•2H_2_O)/meV	∆*Q*
3.16	73.52	−0.01 e

**Fig. 5:**
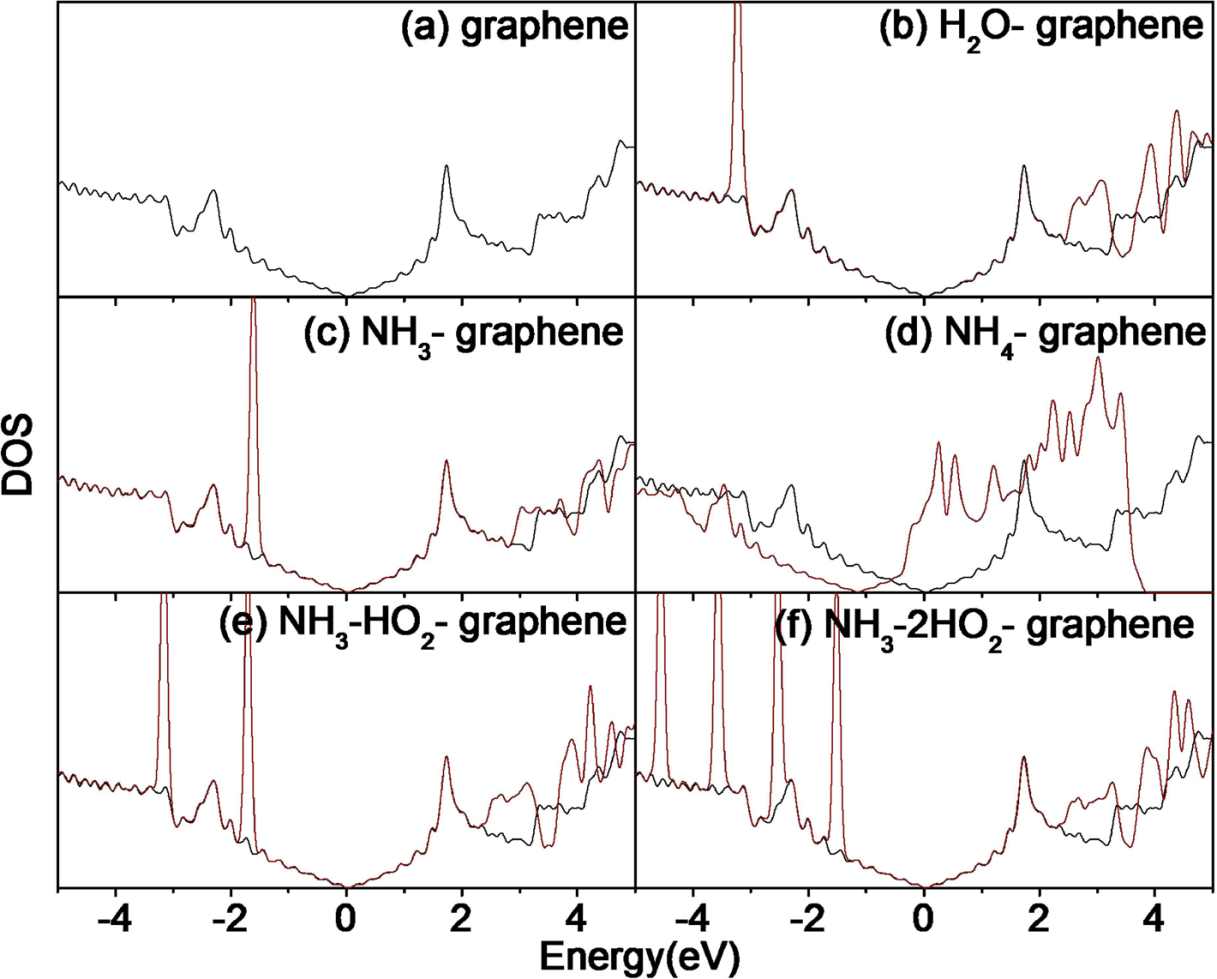
The DOS of the gas-graphene system. Electronic density of state (DOS) of intrinsic graphene (**a**) and the gas adsorbed on graphene (**b–f**, *red line* is the DOS of the gas-graphene system)

To further determine the effects of gas absorption on electrical conductivity, the electronic densities of state (DOS) for all of the systems are calculated in Fig. 5. Figure 5b, c shows that the DOS of H_2_O-graphene system and NH_3_-graphene system at the Fermi level changes little between molecules and graphene alone, resulting from the weak van der Waals interactions and the small charge transfer. The data depicted in Fig. 5d demonstrate that reaction with NH_4_ is an effective way of molecular doping of graphene. The attachment of one NH_4_ group per 18 carbon atoms gives rise to a shift of the DOS so that the Fermi level of the NH_4_-laden system lies within the conduction band. In other words, NH_4_ physisorbed impurities act as strong donors for the basal plane of graphene. Figure 5e, f shows the group of NH_3_•H_2_O and NH_3_•2H_2_O interacting with graphene sheets with weak force, which proves that the clusters desorption is easier than that of NH_4_ group from graphene films.

## Conclusions

In conclusion, the adsorptions of ammonia on graphene were investigated with experiment and first-principle calculation. The study demonstrates that the remarkable variation of the electrical conductivity is induced by the ammonia adsorption, and that the graphene gas sensor possesses an excellent characteristic of high sensitivity for ammonia gas detection. It was found experimentally that ammonia molecules produced by a bubble system of ammonia water were strongly adsorbed onto the graphene sheets; however, ammonia molecules were only weakly adsorbed onto the intrinsic graphene with small binding energy value and large distance between the NH_3_ molecules and graphene from first-principle calculations. The electronic structure and electrical conductivity of the intrinsic graphene have limited changes due to the adsorption of NH_3_ molecules. Moreover, this study found that the ammonium (NH_4_) had strong interactions with graphene, forming a strong bond that introduces a large amount of shallow donor states into the system. When adding water molecules into the desorption process, the whole desorption process was greatly accelerated. We considered that ammonium molecules and water molecules generated the ammonia-water cluster and found that the cluster has weak adsorption with graphene sheets by calculation. The results are in accordance with the experiment. In a word, this study demonstrated that the ammonia gas produced by a bubble system of ammonia water were mainly molecular groups of NH_3_ and NH_4_, and the NH_4_ moleculars are ideal candidates for the molecular doping of graphene. However, the NH_3_ moleculars have weak interaction with graphene.
